# Platelets and Smooth Muscle Cells Affecting the Differentiation of Monocytes

**DOI:** 10.1371/journal.pone.0088172

**Published:** 2014-02-14

**Authors:** Michelle W. Y. Williams, Ann K. Guiffre, John P. Fletcher

**Affiliations:** Department of Surgery, University of Sydney, Westmead Hospital, Westmead, New South Wales, Australia; Keio University School of Medicine, Japan

## Abstract

**Background:**

Atherosclerosis is characterised by the formation of plaques. Monocytes play a pivotal role in plaque development as they differentiate into foam cells, a component of the lipid core whilst smooth muscle cells (SMC) are the principal cell identified in the cap. Recently, the ability of monocytes to differentiate into a myriad of other cell types has been reported. In lieu of these findings the ability of monocytes to differentiate into SMCs/smooth muscle (SM)-like cells was investigated.

**Method and Results:**

Human monocytes were co-cultured with platelets or human coronary aortic SMCs and then analysed to assess their differentiation into SMCs/SM-like cells. The differentiated cells expressed a number of SMC markers and genes as determined by immunofluorescence staining and quantitative polymerase chain reaction (qPCR). CD array analysis identified marker expression profiles that discriminated them from monocytes, macrophages and foam cells as well as the expression of markers which overlapped with fibroblast and mesenchymal cells. Electron microscopy studies identified microfilaments and increased amounts of rough endoplasmic reticulum indicative of the SM- like cells, fibroblasts.

**Conclusions:**

In the appropriate environmental conditions, monocytes can differentiate into SM-like cells potentially contributing to cap formation and plaque stability. Thus, monocytes may play a dual role in the development of plaque formation and ultimately atherosclerosis.

## Introduction

In Australia, an estimated 45,600 people died from cardiovascular disease (CVD) in 2011 representing 31% of deaths. The leading cause of CVD is atherosclerosis, a chronic inflammatory disease where the accumulation of lipids, inflammatory cells and foam cells lead to the development of atherosclerotic plaques. The potential of a plaque to rupture is determined by its stability which in turn is determined by the ratio of the lipid core to the fibrous cap, the more stable plaques containing a larger fibrous cap [22].

Monocytes are known to play a pivotal role in plaque development. These cells ingest excess lipid to form foam cells leading to the production of a lipid core, they also express effector molecules that are pro inflammatory, cytotoxic, chemotactic and release metalloproteinases (MMPs) that degrade the fibrous cap. This ultimately leads to plaque destabilisation and potential rupture [38].

Vascular SMCs (VSMC) are the cells traditionally associated with the fibrous cap. Growth factors and cytokines induce their proliferation and migration from the tunica media into the intima ultimately resulting in fibrous cap formation of the plaque. Studies have since shown that the SMCs/SM-like cells found in the cap could be VSMCs, fibroblasts and/or myofibroblasts [10], [13].

Over the last decade there have been several studies which have reported that other cells aside from medial SMCs contribute to the development of the fibrous cap. Bone marrow progenitor cells were found to be the predominant cell in the neointima of atherosclerotic plaques. Circulating progenitor cells injected into atherosclerotic mice increased SMC accumulation and collagen whilst decreasing macrophage infiltration in late atherosclerotic plaques [47]. CD68 positive cells (monocyte myeloid marker) co-expressing CD34 (haemopoietic marker), collagen I as well as alpha SM actin (fibrocytes) were found in the fibrous cap and neo-collagen rich areas of carotid endarterectomy specimens [25]. In atherosclerosis monocytes may have a dual role in plaque morphology and participate not only in the formation of the lipid core but also the fibrous cap. Thus, these cells potentially have an atheroprotective role via the induction of plaque stability.

Although monocytes are traditionally known to differentiate into macrophage/macrophage foam cells more recently they have been shown to differentiate into numerous other cell types. To date, research groups have cultured CD14+ monocytes and with the aid of growth factors have induced their differentiation into endothelial cells, epithelial cells, hepatocytes, keratinocytes, neuronal cells, adipocytes, osteoblasts, T cells, fibrocytes and myofibroblasts [3], [5], [6], [20], [26], [44], [45]. Similarly, studies have also reported the use of co-culture systems to induce monocytes to differentiate into neural cells, SM-like cells and cardiomyocytes [19], [23]. Thus, CD14+ monocytes have the potential to function like progenitor cells as these studies have shown them to have a pluripotent nature.

More recently, it has been shown that monocyte derived fibrocytes (co-expression of CD34 and collagen I) were identified in the fibrous caps of atherosclerotic plaques [25]. The primary objective of this study was to develop an *in vitro* model representative of an atherosclerotic environment. The secondary objective was to assess the potential of monocytes to further differentiate into SMC/SM-like cells via fibrocytes.

To mimic an atherosclerotic environment human aortic SMCs were selected as in atherosclerosis there is an ongoing wound healing process that occurs whilst the damaged endothelium is being repaired. As part of this process, SMCs/myofibroblasts create the fibrous cap that encapsulates the plaque and separates the thrombotic components of the lipid core from the blood [23].

Platelets were also selected as they play a major role in wound healing by providing the haemostatic plug (part of the initial process of inflammation), which occurs when the endothelium of the arteries are damaged [43]. The platelets produce several growth factors and cytokines responsible for cell differentiation including; Platelet Derived Growth Factor beta beta (PDGFBB), transforming growth factor beta-1 (TGFβ1), epithelial growth factor (EGF), vascular endothelial growth factor (VEGF), basic fibroblast growth factor (bFGF) and insulin growth factor (IGF) [15].

The differentiation of monocytes into SMC/SMC-like cells was investigated using changes in protein and molecular expression as well as differences in cellular structure.

## Methods

Ethics statement for obtaining the monocytes and platelets: Written consent was obtained for each cell sample and was ethically approved by the Western Sydney Local Health District Ethics Committee. Ethics number: JH/JL HREC2004/4/4.11 (2839).

The donation of the HcaSMCs was a cell line from ATCC, catalogue number: ATCC® CRL-1999.

### Cell Culture Systems

#### Isolation of monocytes and platelets

Peripheral blood was collected from healthy volunteers. Consent was provided by each volunteer and approval for the study was granted by the Western Sydney Local Health District Research Ethics Committee. The platelets were obtained by centrifuging sodium citrate collection tubes at 120g for 10 minutes and the platelet rich plasma (PRP) collected. The PRP was centrifuged at 1550g, the supernatant removed and the platelets resuspended in PBS (calcium and magnesium free) (Invitrogen, NY USA). Platelet activation was confirmed by flow cytometry for increases in CD62P expression (data not shown). The remaining blood was diluted with the same volume of PBS, underplayed with Lymphoprep™ 1.077 (Nycomed Pharma AS, Olso, Norway) and spun at 300g. The buffy layer was then collected and monocytes isolated using the CD14 MACS microbeads (Miltenyi Biotec, Germany) system. The average monocyte purity obtained using this isolation method as assessed by FACScan flow cytometer (Becton Dickinson, NJ, USA) was >95%.

Human Coronary Aortic Smooth Muscle Cells (HcaSMC) were grown in smooth muscle supplement media and 10% FBS (Invitrogen, NY, USA).

#### Co-culture models

Monocytes were co-cultured with HcaSMC (M+SMC) or platelets (M+P) with their respective controls being cultured monocytes using the conditions described below. All cultures were incubated at 37°C and 5% CO_2_.

#### Monocytes

Monocytes were all cultured on a fibronectin (5 µg/cm^2^) coated surface in DMEM with the addition of 20 IU/ml penicillin G, 20mg/ml streptomycin, 2mM L-glutamine (Invitrogen, NY, USA) and 10% autologous serum at a concentration of 2.5×10^5^ cells/600 µl of media.

#### Cultured HcaSMC (M+SMCs)

HcaSMC were placed in a 0.4 µm pore Transwell (Corning Costar, MA, USA) at a final concentration of 5×10^3^/100 µl of media to give a final ratio of monocytes to SMC of 50∶1.

#### Cultured Platelets (M+Ps)

Platelets were prepared at a final concentration of 2.5×10^5^ cells/10 µl of PBS and placed in a 0.4 µm pore Transwell containing 90 µl media to give a final ratio of monocytes to platelets of 1∶800.

### Immunofluorescence and Confocal Analysis

For immunofluorescence, the monocytes were cultured on fibronectin coated glass coverslips which were inserted into the tissue culture plates. On days 7 and 14 the cells were fixed with absolute methanol (Sigma Aldrich, St Louis, MI, USA).

#### Immunofluorescence staining

The Transwells were removed and the monocytes rehydrated by the addition of Tris-Buffered Saline (TBS) (Sigma Aldrich, St Louis, MI, USA) for 5 minutes. The coverslips were then removed from the cell culture plates and placed on Superfrost® -Plus poly-L-lysine coated slides (Menzel- Glaser, Saarbrückener, Germany) after which they were stained.

The primary antibodies (Table 1) or isotype controls were incubated with the cells for 30 min at room temperature (RT) and washed gently with TBS three times. Three drops of secondary antibodies (Table 1) was then added for 60 minutes at RT after which the cells were washed again three times with TBS. When double staining was performed the second antibody was added for 30 minutes at RT after which the washing step was performed. Their respective secondary antibodies were then added for 60 minutes at RT after which the cells were washed again three times with TBS. All cultures were then stained with a 1/10,000 dilution of 4',6-diamidino-2-phenylindole (DAPI) (Invitrogen, Grand Island, NY, USA) in PBS for 10 minutes to allow visualization of the nuclei. The stained cells were mounted with aqueous fluorescent mounting media (DAKO, Sydney, Australia).

**Table 1:** List of antibodies used for staining. (N/A =  no designation for clone).

Antibody	Clone	Isotype	Host	Dilution	Supplier
CD34	H-140	IgG	Rabbit	1∶50	Santa Cruz
Procollagen I	M-58	IgG	Rat	1∶200	Santa Cruz
α-Smooth Muscle Actin	αsm-1	IgG2a	Mouse	1∶50	Novocastra
Calponin	CALP	IgG1	Mouse	1∶50	DAKO
Mouse IgG1	Universal	N/A	Mouse	1∶50	DAKO
Rabbit Universal IgG	Universal	N/A	rabbit	neat	DAKO
Rat IgG1k	KLH-GI-2-2	N/A	rat	1∶200	Southern Biotec
Goat anti-rabbit Alexa Fluor 594	N/A	IgG	Goat	1∶250	Molecular Probes
Goat anti -mouse Alexa Fluor 488	N/A	IgG	Goat	1∶250	Molecular Probes
Goat anti-rat Alexa Fluor 488	N/A	IgG	Goat	1∶250	Molecular Probes

All staining was conducted with appropriate positive controls, KG1 leukaemic cell line [33], [43] for CD34 and human umbilical smooth muscle cells [35], [37] for all other markers.

#### Image analysis

Single stained cells were viewed with a Leica DMLB fluorescent microscope (Leica Microsystems, Sydney, Australia) and photos taken using a SPOT camera (RTKE Diagnostic Instruments Inx, MI, USA). Double stained cells were observed under the Olympus 1x81 confocal microscope and Images acquired using the Olympus Fluorview FV1000 (Olympus Australia Pty Ltd, VIC, Australia).

### Quantitative PCR

#### qPCR protocol

RNA was isolated using either the Absolutely RNA Nanoprep Kit (Stratagene, TX, USA) or the Qiagen RNeasy Minikit (Qiagen, Hilden, Germany). Total RNA was transcribed into cDNA using the Affinity Script kit (Stratagene, TX, USA). qPCR was performed using the Brilliant II Sybr Green kit (Stratagene, TX, USA) and samples were set up on the Stratagene Mx 3005P. Controls containing all constituents except reverse transcriptase were performed to rule out contamination by DNA and all constituents except the template to rule out contamination of the reagents. Table 2 represents all the primers that were used.

**Table 2:** Primers used for Real Time PCR.

Primer Name	Primer Sequence	Primer Sequence	Product Size (bp)	Accession Numbers
	(Forward 5′-3′)	(Reverse 5′-3′)		
αSM actin	AAGGCCAACCGGGAGAAAAT	GATGGGGAATTGTGGGTGA	158	NM_001613
Collagen I (a)	ATGCCTGGTGAACGTGGT	CAGGTTGGCCGTCAGCAC	285	NM_000088
CD34	TTGACAACAACGGTACTGCTAC	TGGTGAACACTGTGCTGATTAC	270	NM_001773
Calponin	CAGTGTGCAGACGGAACTCAGCC	GCCGTCCATGAAGTTGTTGC	241	NM_001299
GAPDH	ACCACAGTCCATGCCATCAC	TTCTAGACGGCAGGTCAGGT	225	NM_002046

#### qPCR Analysis

The Stratagene MxPro software determined the average copies of a gene from the duplicate samples which were then normalised against the reference gene GAPDH.

### Cluster of Differentiation (CD) Array

A CD array, containing a panel of 148 antibodies and their respective isotype controls, was performed on monocytes, macrophages, macrophage foam cells, M+Ps and M+SMCs.

### Cell Culture

#### Monocytes

WBCs were isolated from leukopaks (Australia Red Cross Blood Service, NSW, Australia) using the Lymphoprep™ 1.077 and monocytes obtained using the CD14 microbeads system.

#### Macrophages and macrophage foam cells

WBCs were isolated from leukopaks (Australia Red Cross Blood Service, NSW, Australia) using the Lymphoprep 1.077™. Macrophages and macrophages foam cells were generated as per the method described in Davies and Gordon 2005 [9].

#### Macrophages

WBCs were cultured with RPMI 1640 (Invitrogen, Grand Island, NY, USA) containing 7.5% donor plasma for 90 minutes at 37°C, 5% CO_2_. The cells were maintained in X- VIVO media (Lonza Australia Pty Ltd, Mt Waverly, VIC, Australia) with 1% donor plasma, media was changed on day 7 and macrophages harvested on day 10.

#### Macrophage foam cells

Macrophages were cultured as described above. On day 9, acetylated low density lipoprotein was added to the cultures at a concentration of 50 µg/mL of RPMI 1640 media containing 10% lipoprotein deficient serum and cultured for 2 days [4]. Foam cells were harvested on day 11 of culture.

#### M+P and M+SMCs

The cultured monocytes were set up as described above. On day 14 the cells were detached using Accutase (Millipore, CS, USA).

#### Analysis of cell marker expression

The cells were then prepared for CD array analysis by suspending them at a concentration of 0.4x 10^7^ cells/mL (as recommended in the manufacturer’s manual) in PBS containing 2% heat inactivated AB serum (Sigma-Aldrich, MO, USA). The cells were incubated on the array slides for 30 minutes (Medisac Pty Ltd, NSW, Australia) and fixed with 4% formaldehyde. Slides were then placed in the DotReader and scanned by the DotScan Software (Medsaic Pty Ltd, NSW, Australia). Once slides were scanned digital images were captured; after which the proportion of cells in the sample expressing the antigen as well as the level of expression was determined. Prior to statistical analysis each marker was normalised against CD44 as per the Medsaic protocol [14].

### Electron Microscopy Analysis

Electron microscopy was used to assess the ultrastructural morphology of monocytes, M+Ps and M+SMCs. On days 1, 3, 5, 7, 10 and 14 of culture; the cells were fixed with 0.5ml Karnovsky’s fixative and resin embedded. Ultrathin sections were prepared (Leica EM UC6, Leica, Germany) and collected on 400 µm thin bar mesh copper grids (Proscitech, QLD, Australia). Each copper gird was stained with uranyl acetate solution (Sigma-Aldrich, MO, USA) and transferred to lead citrate, washed and allowed to dry prior to electron microscopy analysis. A CM 10 transmission electron microscope (Phillips, NSW, Australia) was used to analyse all stained samples and images were captured on Kodak Electron Microscopy Film 4489 (Holgate Scientific Pty Ltd, NSW, Australia). A minimum of 30 cells from 2 different sections were analysed for each sample.

### Statistical Analysis

All statistical analysis were performed using the Statistical Product and Service Solutions Version 17 (SPSS Inc, Chicago, USA) (SPSS).

qPCR statistical analysis: GAPDH was used to normalize the data and differences were analysed using the Student’s T Test. All tests were two tailed and differences were considered to be significant, *P<0.05. Expression of fold increases in gene expression was analysed by comparing the M+P or M+SMCs against the monocytes.

CD Array statistical analysis: All data were analysed using the two-tailed tests. The expression levels were log transformed to approximate normality prior to analysis. Analysis of variance was used to test for heterogeneity in log expression levels for each cell surface marker in each cell type. To place the highest stringency on the analysis of the data P<0.001 was chosen. This p value ensures that any cell surface markers that are significant would be 99.9% assured.

## Results

### Expression of Markers by M+SMCs, M+Ps and Cultured Monocytes by Immunofluorescence and CD Array

**Figure 1:**
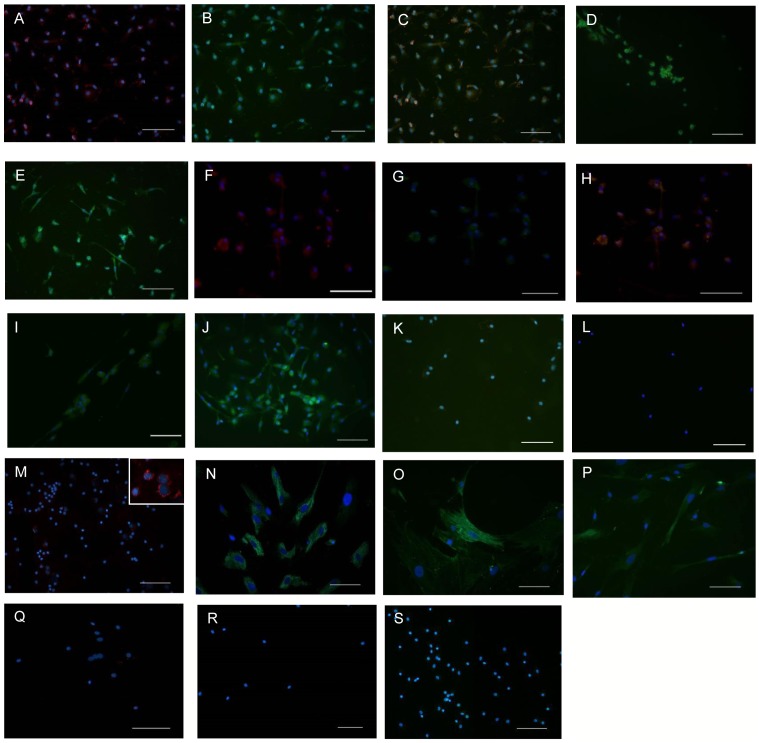
Immunofluoresence staining of M+SMCs, M+Ps and Monocytes on day 14 of culture. ***M+SMCs*:** A) CD34 (red), B) Procollagen I (green), C) the expression of double staining of CD34 (red cell surface) and procollagen I (green cytoplasm). D) αSM actin and E) calponin (both green). ***M+P:*** F) CD34 (red), G) Procollagen I (green), H) positive expression of both CD34 and procollagen I (orange). I) αSM actin and J) calponin (both green). ***Monocytes:*** lack of expression of αSMC actin and calponin (K & L respectively). ***Positive controls:*** M) CD34 (red), N) Procollagen I (green), O) αSMC actin (green), P) Calponin (green). ***Isotype controls:*** (Q) rabbit IgG, (R) mouse IgG and (S) rat IgG. Nuclei of the cells are blue. Scale bars 100 µm.

To study the differentiation potential of monocytes into smooth muscle like cells, monocytes were co-cultured with platelets or HcaSMCs and analysed for cell marker expression. The M+SMCs co-expressed CD34 and procollagen I (Figure 1C), αSM actin (Figure 1D) from day 7 onwards and calponin from day 14 (Figure 1E). The M+Ps co-expressed CD34 and procollagen I (Figure 1H) from day 7 onwards, whilst the SMC markers; αSM actin (Figure 1I) and calponin (Figure 1J) were expressed from day 14 onwards. Monocytes cultured without SMCs or platelets did not express any of the SMC markers (Figure1 K&L).

Analysis by CD array of surface markers identified a number of significant differences between the co-cultured cells and those cells typically found in atherosclerotic plaques. A comparison of M+SMC to monocytes, macrophages and foam cells found that M+SMC could be distinguished from monocytes by their significantly lower levels of CD38 and CD182; from macrophages by their significantly lower levels of CD81 and CD254 and from foam cells by their significantly lower levels of CD61, CD81, CD269 and CD281. A comparison of M+P found that these cells could be distinguished from monocytes by their significantly lower levels of CD11a, CD33, CD38, CD52, CD95, CD181, CD182, and their significantly higher levels of CD29 and CD71; from macrophages by their significantly lower levels of CD11a, CD11c and CD254 and from foam cells by significantly lower levels of CD2, CD10, CD11a, CD16, CD22, CD33, CD37, CD40, CD45RA, CD281 and significantly higher levels of CD5. Table 3 represents the statistically significant marker expression of each cell when compared to one another. There were no significant marker differences between M+SMC and M+P cells. When comparing the co-cultured monocytes with monocytes, macrophages and foam cells the results showed that the co-cultured monocytes expressed the myeloid markers including CD14 and CD45 at levels that were not significantly different to monocytes, macrophages and foam cells.

**Table 3:** CD surface markers expressed by each cell type.

	Monocytes		Macrophages		Macrophage foam cells		M+SMCs		M+Ps	
*Monocytes*			CD71	16.21 fold (CI 9.57–27.46)	CD80	65.38 fold (CI 52.29–81.74)	CD281	1.95 fold(CI 1.59–2.39)	CD71	16.6 fold (CI 5.38–51.11)
			CD54	14.2 fold (CI 7.37–27.36)	CD22	47.39 fold (CI 29.47–76.20)			CD29	2.3 fold (CI 1.66–3.29)
			CD254	13.51 fold (CI 6.15–29.69)	CD37	44.45 fold (CI 28.57–69.10)				
			CD9	5.64 fold (CI 3.53–9.01)	CD281Ser	43.31 fold (CI 22.56–83.15)				
					CD71	23.92 fold (CI 10.60–53.99)				
					CD182	21.22 fold (CI 9.40–47.90)				
					CD54	16.21 fold (CI 9.57–27.46)				
					CD281AB	11.36 fold (CI 4.09–31.60)				
					CD45RA	8.82 fold (CI 3.46–22.52)				
					CD254	7.03 fold (CI 4.59–10.78)				
					CD9	7.01 fold (CI 4.67–10.52)				
					CD278	1.84 fold (CI 2.38–1.43)				
*Macrophages*	CD182	53.26 fold (CI 23.89–118.73)			CD281Ser	26.03 fold (CI 9.87–69.86)				
	CD181	17.22 fold (CI 6.22–47.68)								
	CD184	1.92 fold (CI 1.69–2.19)								
	CD124	1.63 fold (CI 1.35–1.95)								
	CD11c	1.59 fold (CI 1.42–1.76)								
*Macrophage foam cells*	CD4	2.74 fold (CI 3.46–22.52)							CD5	5.76 fold (CI 10.34–3.21)
	CD64	1.33 fold (CI 1.18–1.50)								
*M+SMCs*	CD38	4.36 fold (CI 2.41–7.90)	CD81	22.88 fold (CI 12.83–40.81)	CD81	24.29 fold (CI 12.62–46.77)				
	CD182	56.43 fold (CI 20.22–157.49)	CD254	16.54 fold (CI 12.83–40.81)	CD281	22.2 fold (CI 10–49.3)				
					CD269	6.76 fold (CI 3.38–13.52)				
					CD61	2.34 fold (CI 1.7–3.2)				
*M+Ps*	CD52	55.5 fold (CI 14.35–214.63)	CD254	6.4 fold (CI 6.14–43.83)	CD33	27.96 fold (CI 7.53–103.85)				
	CD33	49.3 fold (CI 13.48–180.16)	CD11a	2.9 fold (CI 1.92–4.36)	CD281	25.59 fold (CI 9.82–66.68)				
	CD181	15.9 fold (CI 6.72–38.02)	CD11c	2.72 fold (CI 1.84–4.00)	CD22	24.26 fold (CI 9.21–63.89)				
	CD95	7.5 fold (CI 4.02–14.14)			CD45RA	20.02 fold (CI 6.94–57.76)				
	CD38	6.5 fold (CI 3.5–11.89)			CD10	19.94 fold (CI 6.05–41.96)				
	CD182	4.4 fold (CI 2.41–7.9)			CD37	17.13 fold (CI 6.29–46.67)				
	CD11a	4.32 fold (CI 3.15–5.12)			CD40	11.86 fold (CI 6.13–22.95)				
					CD2	7.67 fold (CI 3.56–16.52)				
					CD16	4.60 fold (CI 3.49–6.06)				
					CD11a	3.53 fold (CI 2.63–4.75)				

The table records all the markers which were significantly (P<0.001) expressed by each cell type listed in the horizontal axis (bold font) when compared to the cell types listed in the vertical axis (italics font) with their associated 95% confidence intervals (CI). For example, monocytes expressed the marker CD182, 53.26 fold significantly higher than macrophages.

### Expression of Markers by M+SMCs, M+Ps and Cultured Monocytes by qPCR

qPCR was used to examine the level of gene expression in the co-cultures M+P and M+SMCs as compared to monocytes.

#### M+SMCs (Figure 2A)

Although not significant there was an increase in αSM actin marker of 1.07 fold (P = 0.53) and 1.19 fold (P = 0.63) on days 7 and 14 respectively. This same increase was also found in collagen I by 1.64 fold (P = 0.47) and 6.34 fold (P = 0.2) on day 7 and 14 respectively. In terms of expression there was a 10.5 fold (P = 0.083) increase of Calponin expression and a 2.25 significant fold (P = 0.005) increase in the expression of CD34. This was reflected in co-cultured monocytes and occurred on day 14.

**Figure 2:**
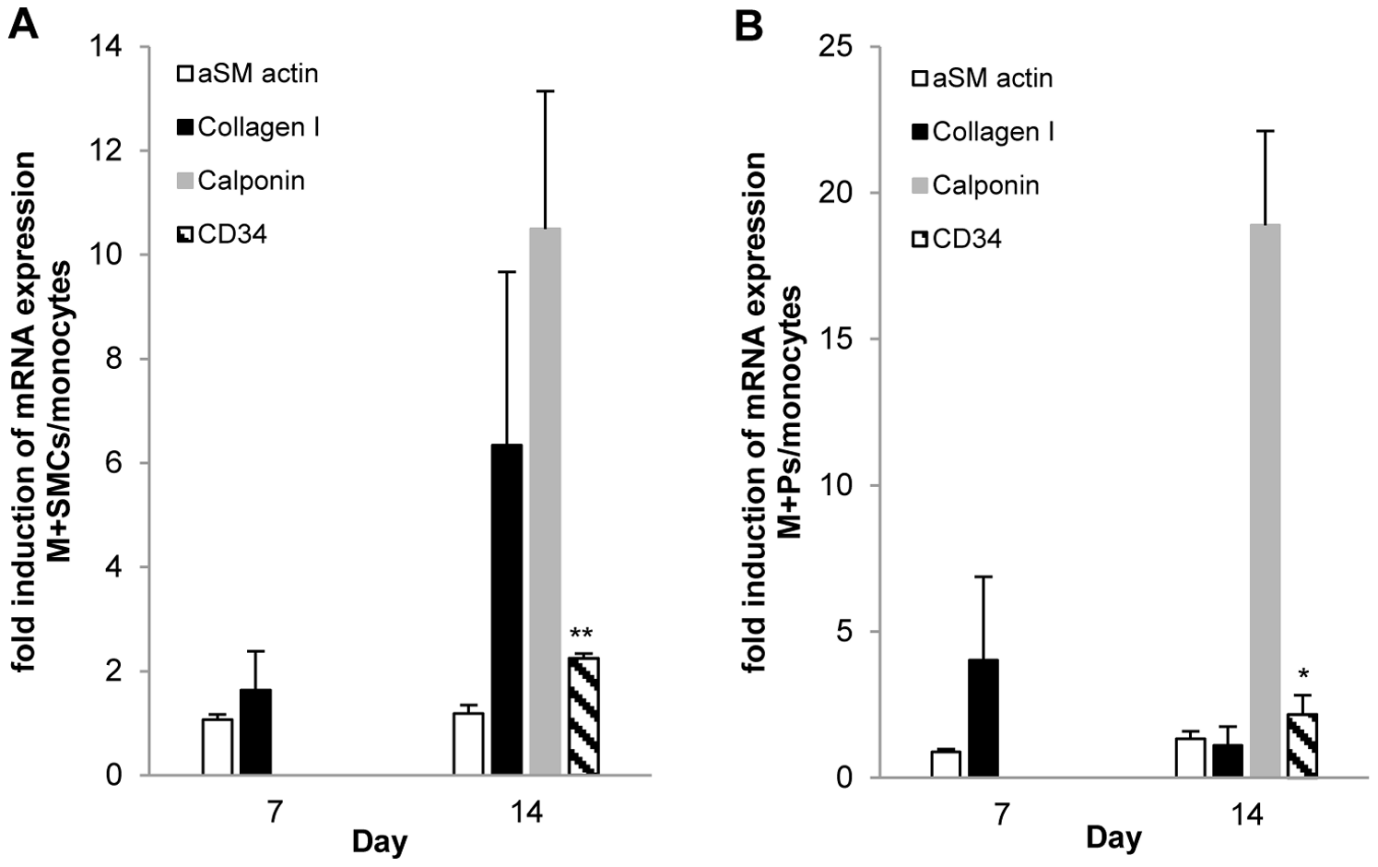
Monocytes co-cultured with HcaSMCs (M+SMCs) and platelets (M+Ps) induce the expression of smooth muscle cell markers analysed by qPCR. A) M+SMCs (n = 3) and B) M+Ps (n = 3) were compared with cultured monocytes for the expression of αSM actin, collagen I, calponin and CD34. Data were normalised to the expression of GAPDH. Statistically significant * denotes P-value<0.05.

#### M+Ps (Figure 2B)

There was an increase in αSM actin of 1.33 fold (P = 0.47), significantly greater level of expression of calponin of 18.9 fold (P = 0.03) and CD34 of 2.16 fold (P = 0.1) on day 14. Although not significant, the level of expression of collagen I was greater in co-cultured monocytes on both day 7 at 4.03 fold (P = 0.48) and day 14 at 1.11 fold (P = 0.9) when compared to monocytes cultured alone.

### Differences in Phenotypic Analysis Comparing M+SMCs and M+Ps from Cultured Monocytes

All cell types were seen to develop azurophilic granules, vacuoles, lysosomes, all had phagocytic abilities and lost their fingerlike projections from day 3 of culture.

**Figure 3:**
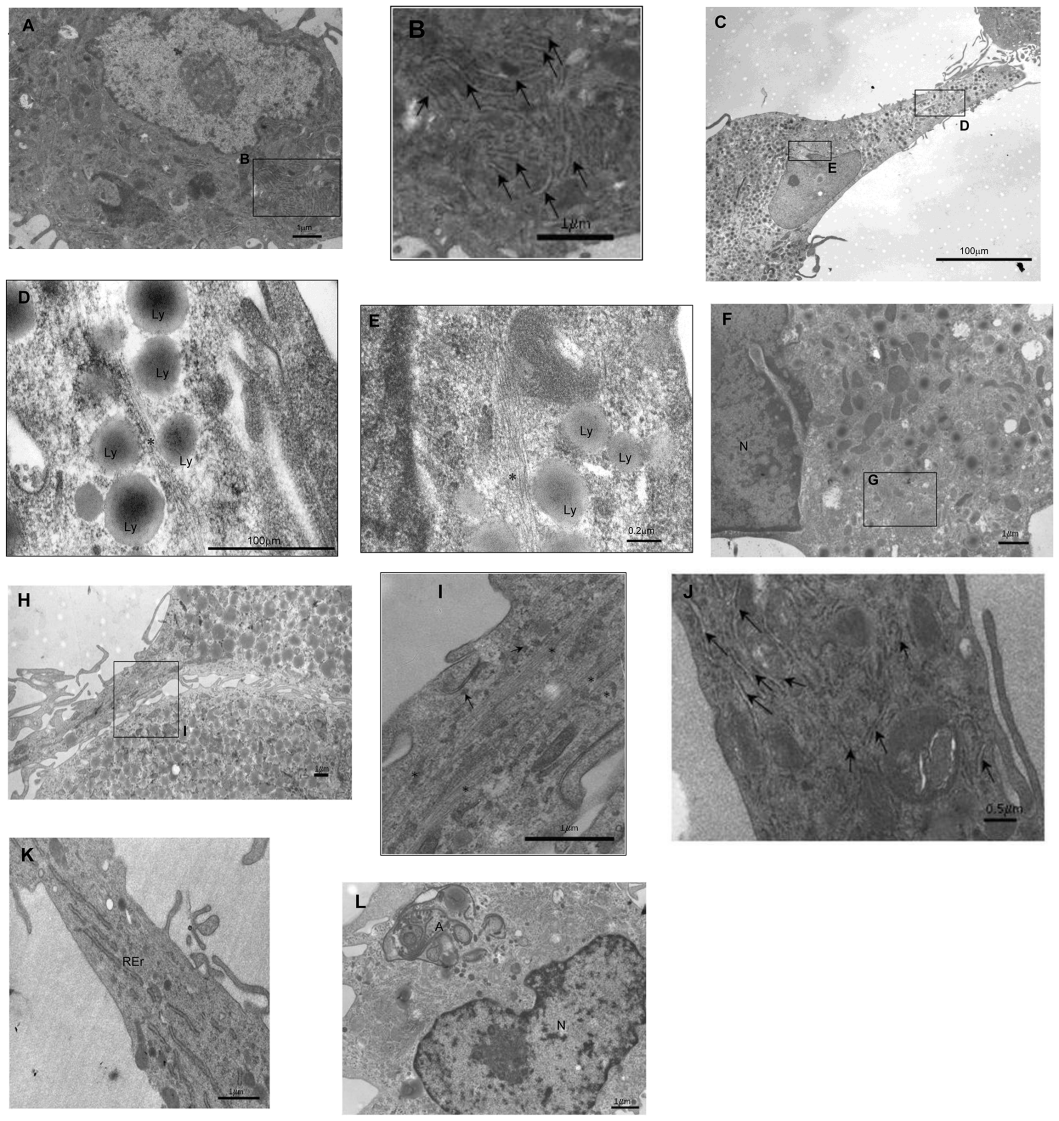
Electron microscopy analysis of M+SMCs and M+Ps. ***M+SMCs*.** Figure A) Day 7 of culture containing ribosomes (arrows) (B). Figure C) Day 10 of culture with microfilaments* surrounded by lysosomes (Ly) (D) and present closer to the nucleus (E) ***M+Ps*** Figure F) Day 7 culture containing microfilaments* (G). H) Day 10 culture containing microfilaments* surrounded by monoribosomes (arrows) (I). Figure J) Day 5, containing rough endoplasmic reticulum (rER) with ribosomes attached (small electron dense beads) (arrows) and day 14 cells showing rER with average length of 1.9 µm (Figure K). ***Monocytes*** Figure L) Day 14 culture with the presence of autophagy (A) and a nucleus (N).

The cells generated from M+SMC cultures were found to have increases in the number of short profiles of rough endoplasmic reticulum (rER) within the cytoplasm of the cells with a length of approximately 0.8 µm on day 7 (Figures 3A&B). On day 10, microfilaments were found in the elongated regions of the cells (Figures 3C-E). The cells generated from M+Ps cultures were found to contain small short rER with an average length of 0.8 µm from day 5 (Figure 3J) and had elongated to an average of 1.9 µm in length by day 14 of culture (Figure 3K). On day 7, small bundles of microfilaments made up of parallel filaments were observed (Figure 3F&G). These were more evident in the cell cytoplasm and located at the elongated region of the cell accompanied by numerous monoribosomes on day 10 (Figure 3H&I).

Three differences were identified between cultured and co-cultured monocytes which indicated that the co-cultured cells had differentiated: Firstly, there was an increase in rER observed in the co-cultured monocytes. Secondly, the co-cultured cells showed the presence of microfilaments. Thirdly, there was the absence of autophagy which was observed only in the monocytes alone (Figure 1L). The increase in the number of rER and the presence of microfilaments located distally from the nucleus within the elongated regions are characteristics of myofibroblasts and fibroblasts.

## Discussion

The role of monocytes in the formation of foam cells in atherosclerosis has been well documented [18]. More recently, the pluripotent nature of monocytes has been reported. This is the first study to report that monocytes in an atherosclerotic setting, when co-cultured with SMCs or platelets further differentiated into cells expressing both fibroblast and myofibroblast characteristics as determined by immunofluorescence, qPCR, CD array and electron microscopy.

For this study a culture model was established as per Abe 2001 [1] where platelets or HcaSMCs were placed in a 0.4 µm Transwell. This Transwell size was selected to ensure that the platelets (13–27 cubic µm [27]) or HcaSMCs (100 µm in length and 5–10 µm in width [39]) could not pass through the Transwell pores into the culture well containing the monocytes. This Transwell model was setup as initial experiments where SMC or platelet conditioned media was cultured with monocytes were found not to induce SMC marker expression (data not shown). This finding suggests that an ongoing release of cytokines by SMCs and platelets (PDGFBB, TGFβ1, EGF, VEGF, bFGF and IGF) [8], [24], [29] is required to induce differentiation in these two models.

Immunofluorescence and qPCR data found CD34 and the markers: αSM actin, collagen I and calponin were expressed by cells in both co-culture systems. All these markers were expressed at levels greater than cultured monocytes as determined by qPCR. The following markers were chosen for the following reasons; αSM actin forms part of the cytoskeleton, plays an essential role in regulating cell movement associated with SMCs [28] and has been used as a marker for SMCs [30]. Collagen I functions to provide the scaffolding and support for a wound during the processes of wound healing [11]. It is needed for the repair of the anatomic structure, strength and function of the injured tissue [36]. Calponin is involved in cell contraction, by inducing actin filament bundling leading to contraction and it is an identifying marker of SMCs [30]. Interestingly, the expression of CD34 by immunofluorescence staining was observed from day 7 in all culture conditions even though mRNA was not detected until day 14 via qPCR. Reports in the literature have noted that low/undetectable levels of mRNA with protein expression can occur [17] and that mRNA can be stored in the nucleus and only released once protein levels have reached a threshold limit [34].

Unexpected observations were found: cultured monocytes expressed both CD34 and Collagen I. These findings are in agreement with Curnow 2010 [7] who also reported that monocytes cultured in serum media expressed these markers.

Immunofluorescence and qPCR results show that cultured monocytes differentiate into fibrocytes (co expression of CD34 and collagen I) irrespective of culture conditions. The findings reveal that the addition of SMCs or platelets promotes further differentiation into SM like cells.

Additional cell surface marker analysis by CD array showed that M+SMCs and M+Ps could be distinguished from their precursor the monocyte, macrophages and macrophage foam cells. Notably, these cells did not express markers known to be involved in the process of atherosclerotic plaque progression by their significant lower expression of CD254 and CD281 when compared to macrophages and foam cells respectively. The expression of CD254 is associated with calcification and plaque rupture [2] and CD281 (Toll- like receptor-1) reflect the activate state of macrophages or foam cells in phagocytosing foreign particles like lipid in the area of atherosclerosis. These markers are found predominantly in areas of damaged endothelium and high inflammatory cell localisation in atherosclerotic plaques where macrophages foam cells reside. The CD array results indicated that the SMCs and platelets provide an environment which enables the monocytes to start differentiating into cells that do not play a role in plaque instability.

Phenotype analysis was conducted using EM. The co-cultured monocytes identified that our cells contained increased numbers of rER indicating a higher level of protein synthesis, characteristic of a myofibroblast/fibroblast [16]. Microfilaments/actin filaments located within the elongated regions of the co-cultured cells are indicative of locomotive. These filaments could function to enable the cell to move around their environment to search and endocytose foreign material in the blood or in tissues for elimination. These two features were not found in cultured monocytes.

These two features are therefore suggestive of a fibroblast cell type, classed as a SM like cell. Our co-cultured monocytes were not representative of mature SMCs as they lacked dense bodies and abundant mitochondria, did not contain myofilaments and contained few filament bundles. They were also not typical myofibroblasts by the absence of the myofilaments as well as fibronexus [42]. The literature reports that mesenchymal cells have similar markers to fibroblasts with many overlapping markers [12]. The increased amounts of microfilaments, rER and the expression of fibroblast markers CD29 and CD81 reported by the CD array is supporting evidence that the co-cultured monocytes have differentiated into a mesenchymal/fibroblast like cell.

The significance of this work shows the capability of a circulating blood monocyte to differentiate into a cell that expresses markers including αSM actin and CD45. These findings are in agreement with a number of *in vivo* studies showing that circulating progenitor cells expressing αSM actin and CD45 are extensively recruited in a variety of atherosclerotic injury models [32], [40], [41]. The αSM actin^+^ CD45^+^ cells in these models were located in the neointimal regions of the injury. These studies support the potential of the cells generated in our model to be involved in atherosclerotic plaques and fibrous cap formation. Further studies are needed to show that circulating monocytes have the ability to localise in atherosclerotic plaques and are able to form fibrous plaques in an *in vivo* model.

In both co-culture systems the environmental conditions provided by the SMCs and platelets resulted in the differentiation of monocytes to SM like cells with mesenchymal/fibroblastic like characteristics. These cells do maintain their myeloid lineage by their marker expression of CD14 but they also expressed collagen I, αSM actin and calponin (found in SMCs) with the additional phenotypic changes. Although CD14 expression is reported to decrease as monocytes mature, it has also been reported that maturing monocytes can maintain their levels of CD14 expression [46] whilst tumour necrosis factor, interleukin -1 and 6 moderately increases CD14 [21], [31]. These studies demonstrate that maturing monocytes can maintain or increase their level of CD14 expression as per the cytokines they were exposed to. Future studies analysing the culture environment provided by the platelets or SMCs would identify the cytokines present in these models. These results support the idea that monocytes in atherosclerosis may be induced to play a greater role in plaque stability. In future studies, the functional and further differentiation abilities of these cells and how these co-cultured cells differ from one another would be determined to consolidate this data.
